# Histological and ultrastructural degenerative findings in the gluteus medius tendon after hip arthroplasty

**DOI:** 10.1186/s13018-021-02434-1

**Published:** 2021-05-26

**Authors:** Mustafa Ibrahim, Urban Hedlundh, Ninni Sernert, Khaled Meknas, Lars Haag, Tomas Movin, Nikos Papadogiannakis, Jüri-Toomas Kartus

**Affiliations:** 1 Department of Orthopedics, Region Västra Götaland, NU Hospital Group, Trollhättan / Uddevalla, Sweden; 2 University of Gothenburg, Gothenburg, Sweden; 3 Institution of Clinical Science, Sahlgrenska Academy, Gothenburg, Sweden; 4 Department of Orthopedics, University Hospital North Norway, N-9038 Tromsø, Norway; 5 Orthopedics Research Group, Institute of Clinical Medicine, the Arctic University of Norway, Tromsø, Norway; 6 Department of Laboratory Medicine, Division of Pathology, Karolinska Institutet, Stockholm, Sweden; 7 Department of Clinical Science, Intervention and Technology, Division of Orthopedics and Biotechnology, Karolinska Institutet, Stockholm, Sweden; 8 Department of Research and Development, NU Hospital Group, Trollhättan / Uddevalla, Sweden

**Keywords:** Gluteus medius, Tendinopathy, Tendinosis, Hip arthroplasty, Hip replacement

## Abstract

**Background:**

Despite gluteus medius (GMED) tendinosis being relatively common, its presence in association with hip osteoarthritis (OA) or total hip arthroplasty (THA) is not well studied. It was hypothesized that more tendon degeneration would be found in patients with OA of the hip and in those that had undergone THA than that in a control group.

**Methods:**

One hundred patients were included between 2016 and 2019 and were included into 4 groups; the patients were undergoing revision surgery in two groups and primary THA in the other two groups; 22 patients had previously undergone primary THA through a direct lateral approach (involving sectioning of the GMED tendon), 24 patients had previously undergone primary THA through a posterior approach (leaving the GMED tendon intact), 29 patients had primary hip OA, and 25 patients who suffered a femoral neck fracture served as controls. Biopsies from the GMED tendon were obtained at the time of the primary THA or the hip revision surgery. The tendon biopsies were examined ultrastructurally and histologically.

**Results:**

Ultrastructurally, the direct lateral and posterior revision groups had statistically significantly more collagen fibrils with smaller diameters compared with the fracture and primary THA groups. Moreover, the direct lateral revision group had more collagen fibrils with smaller diameters compared with the posterior revision group.

Histologically, the direct lateral revision group had a higher total degeneration score (TDS) compared with the primary hip OA group.

**Conclusions:**

The GMED tendon shows more ultrastructural degeneration in patients who undergo hip revision arthroplasty than in patients with primary OA of the hip and control patients, who had suffered a femoral neck fracture. Furthermore, patients who had previously undergone primary THA through a direct lateral approach revealed more histological GMED tendon degeneration than patients who suffer primary hip OA.

**Supplementary Information:**

The online version contains supplementary material available at 10.1186/s13018-021-02434-1.

## Background

Even if the beneficial effects of total hip arthroplasty (THA) for treatment of osteoarthritis (OA) have been documented, more than one in ten patients are not entirely satisfied [1]. There are complications like infections, implant loosening, wear, and dislocations requiring revision surgery, but unexplained pain and limping are additional common complaints after both primary and revision surgeries [2]. Limping is associated with abductor muscle insufficiency, a symptom of OA, and also a pronounced dissatisfaction outcome measure after hip arthroplasty. As a result, there is a desire for increased knowledge of the gluteus medius (GMED) tendon regarding both the histological appearance and ultrastructural changes.

Traditionally, the two most commonly used approaches have been the direct lateral and the posterior [3]. The direct lateral approach necessitates the partial surgical release of the GMED tendon at its insertion on the greater trochanter [4]. The GMED tendon is spared in the posterior approach. However, this approach has been shown to involve an increased risk of postoperative dislocations [5–7].

Advocates of the posterior approach have emphasized the positive clinical results of sparing the abductor muscles. Patients have been presented with higher mean satisfaction values [8], a greater improvement in function evaluated with the Oxford Hip Score [9] and considerably better outcomes 1–3 years after THA in terms of self-reported limping [10]. However, this has not been confirmed in a meta-analysis, regarding visual analogue scale (VAS) pain and Harris Hip Scores [11].

Limping may be caused by progressive damage to the GMED muscle and tendon [5]. This tendon pathology also appears to progress on MRI from tendinosis to low-grade partial tears and further to high-grade partial tears with corresponding muscle atrophy [12]. Furthermore, in a recently published study, asymptomatic gluteal tendinopathies have been shown to have a negative effect on the outcomes of THA [13].

The internal obturator tendon in patients with OA of the hip has been shown to have a more degenerative appearance compared with those without OA [14]. Similar findings have been reported in the shoulder and to some extent in the knee [15, 16].

Tendon degeneration leads to the formation of collagen III fibrils, which have a smaller diameter than collagen I fibrils, the main collagen type in tendon tissue. The distribution of fibril diameters in tendinopathic tendons thus exhibits a shift towards smaller diameters [17].

The hypothesis of the study was that more GMED tendon degeneration would be found in patients who undergo revision hip arthroplasty than that in patients who undergo primary THA due to OA of the hip or femoral neck fracture. In addition, more GMED tendon degeneration would be found in patients who had previously undergone primary THA through a direct lateral approach than in patients who had previously undergone THA through a posterior approach.

The primary variable in the study was the fibril diameter in the GMED tendon, as seen under the electron microscope.

## Methods

The aim of this case–control study was to investigate the histological and ultrastructural changes to the GMED tendon and to determine whether OA and previous implant surgery lead to more degeneration in the tendon.

A total of 100 patients participated in the study and underwent surgery between 2016 and 2019. These patients were included in one of four groups; the direct lateral revision group included patients who were scheduled for hip revision arthroplasty and had previously undergone primary THA through a direct lateral approach (involving sectioning of the GMED); the posterior revision group included patients who were scheduled for hip revision arthroplasty and had previously undergone primary THA through a posterior approach (leaving the GMED tendon intact); the primary OA group included patients who were scheduled for primary THA due to OA of the hip, and lastly, the fracture group included patients who were scheduled for primary THA due to femoral neck fracture (Table 1).

**Table 1:** Demographics of patients in the study groups

	Revision DL	Revision posterior	Primary THA (OA)	Primary THA (fracture)
*n*	22	24	29	25
Age, mean (SD)	73.4 (10.85)	75.6 (7.67)	70.0 (9.84)	72.8 (5.09)
Female	10	11	19	19
Male	12	13	10	6
*p* value, gender	n.s. (0.756)			
Time between primary & revision surgeries (years)				
Mean (SD)	11.5 (7.1)	14.8 (6.7)		
Median (range)	11.5 (1–23)	13.5 (1–29)		

Revision DL: revision arthroplasty via the direct lateral approach; Revision posterior: revision arthroplasty via the posterior approach; Primary THA (OA): primary total hip arthroplasty due to osteoarthritis; Primary THA (fracture): primary total hip arthroplasty due to femoral neck fracture

*n.s.* not significant, *n* number of patients, *SD* standard deviation

One patient underwent revision surgery due to the failure of metal-on-metal hip resurfacing arthroplasty, two patients due to recurrent hip prosthesis dislocation, while the rest were due to loosening of the hip prosthesis.

The inclusion criteria for both the direct lateral and posterior revision groups were patients with an indication for hip prosthesis revision surgery due to prosthesis loosening, wear, or repeated dislocations. For the primary THA group, the inclusion criterion was primary OA of the hip, while, for the fracture group, it was a displaced non-pathologic femoral neck fracture without OA of the hip. The exclusion criteria were secondary hip arthritis, previous hip surgery (other than primary THA), diseases or conditions that affect the neuromuscular function of the lower extremities (polio, stroke, multiple sclerosis, etc.), osteonecrosis of the femoral head, fragile patients with multiple illnesses or severely ill patients, dementia or cognitive impairment, widespread malignancy, and systemic corticosteroid treatment for more than 3 months. In addition, for the direct lateral revision and posterior revision groups, patients with hip dysplasia, a postoperative infection after the primary THA, and revision < 1 year after the primary THA were excluded.

The material in this case–control study consisted of samples from the GMED tendon, obtained in an open fashion at the time of the primary THA due to femoral neck fracture or OA, as well as at hip revision arthroplasty. The GMED tendon was easily accessible during these operations. Four samples were obtained from each patient. Each biopsy was about 0.5 × 0.5-cm large and was harvested at the insertion site on the trochanter major. The first author obtained all the biopsies in the fracture group, while three other senior orthopedic surgeons obtained all the biopsies in the other groups.

### Histological analysis

The samples for light microscopy were fixed in 10% neutral-buffered formalin, embedded in paraffin blocks and sectioned at 4–5 μm. The sections were stained with hematoxylin–eosin (HE) to evaluate the fiber structure, cellularity, and vascularity. Alcian blue (pH 2.5)-periodic acid Schiff (AB/PAS) was used for the detection of glycosaminoglycan (GAGs)-rich areas. The histological evaluations of two samples from each patient were performed by a pathologist (N.P.) and an orthopedic surgeon (T.M.) with a special interest in pathology together using a light microscope (Leica DMRBE, Wetzlar, Germany). The examiners were blinded in terms of the group to which the patient belonged.

The fiber structure, cellularity, and vascularity and the presence of GAGs were classified according to a semi-quantitative scoring system (Table 2) [15, 16]. It consists of four different elements. Each element can obtain between 0 and 3 points. This procedure and evaluation system has been utilized in multiple previous studies [14, 18–21]. Subsequently, the total degeneration score (TDS) was calculated by adding the mean values of the two biopsies for the four elements. The TDS can result in values between 0 (no degeneration at all) and 12 points (extremely high degeneration). The TDS is similar to a scoring concept previously described and used in a biopsy analysis of the Achilles tendon. The score has also undergone satisfactory intra-observer reliability testing [22].

**Table 2:** Evaluation of biopsy samples with a semi-quantitative four-point scoring system

	Grade 0	Grade 1	Grade 2	Grade 3
Fiber structure	Straight, parallel, packed fibers, with slight waviness	Slight separation of fibers, increased waviness	Separation of fibers, deterioration of fibers	Complete loss of fiber structure and hyalinization
Cellularity	< 100 cells/high-power field (HPF)	100–199 cells/HPF	200–299 cells/HPF	> 300 cells/HPF
Vascularity	Vessels running parallel to the collagen fiber bundles in the septa	Slight increase in vessels, including transverse vessels in the tendon tissue	Moderate increase in vessels within the tendon tissue	Markedly increased vascularity with clusters of vessels
Glycosaminoglycans	No alcianophilia	Slight alcianophilia between the collagen fibers	Moderate increase in alcianophilia	Markedly increased alcianophilia forming blue lakes

### Ultrastructural analysis

Specimens were collected and immediately fixed in 2% glutaraldehyde and 1% paraformaldehyde in 0.1 M sodium cacodylate buffer containing 0.1 M sucrose and 3 mM CaCl_2_ (pH 7.4) at room temperature for 30 min, followed by storage at 4 °C. The specimens were rinsed in 0.1 M sodium phosphate buffer (pH 7.4) prior to post-fixation in 2% osmium tetroxide in 0.1 M sodium phosphate buffer (pH 7.4) at 4 °C for 2 h. The specimens were then dehydrated stepwise in ethanol, followed by acetone and LX-112 (Ladd) embedding. Ultrathin sections (approximately 60–80 nm) were prepared and contrasted with uranyl acetate followed by lead citrate and examined in a Tecnai G2 Spirit BioTWIN electron microscope (FEI) operated at 80 kV and equipped with a 2kx2k Veleta CCD camera (Olympus Soft Imaging System). Four randomly acquired images in areas showing transversely sectioned collagen fibrils were used for image analysis and fibril diameter measurement. The fibril diameters were measured manually on images acquired at × 49.000 magnification (1.14 nm/px) using Fiji software (https://imagej.net/ImageJ) and the Bio-Formats plugin.

The fibrils were grouped in intervals of 10 nm and presented as the relative distribution. One hundred fibrils were analyzed in each specimen, and the mean value was calculated with an accuracy of 1/10th of a nanometer. Two biopsy specimens from each patient were scanned; however, the fibril diameters were only measured in the biopsy with the best transverse orientation, while the other biopsy was left unmeasured. The micrographs were evaluated by one independent technician (L.H.) with extensive experience of using the transmission electron microscope (TEM), and the technician was blinded to the group of specimens.

### Statistical analysis

Median (range) and mean (SD) values are presented for the TEM findings. For the histological findings, a stratified distribution is presented. First, the ANOVA test and the Kruskal–Wallis test were used to test all four groups for the parametric and non-parametric variables, respectively. Subsequently, the unpaired *t* test and the Mann–Whitney *U* test were used for comparisons of the fibril diameters and the histological findings of the TDS respectively between the study groups. The power analysis was based on the assumption that it would be meaningful to detect a difference of 5 nm in fibril diameter between the study groups. If the SD were as large as 40 nm, just over 1000 fibrils would need to be measured to reach a power of 80%. The value of alpha used in the power analysis was 0.05.

## Results

### TEM evaluation

**Fig. 1:**
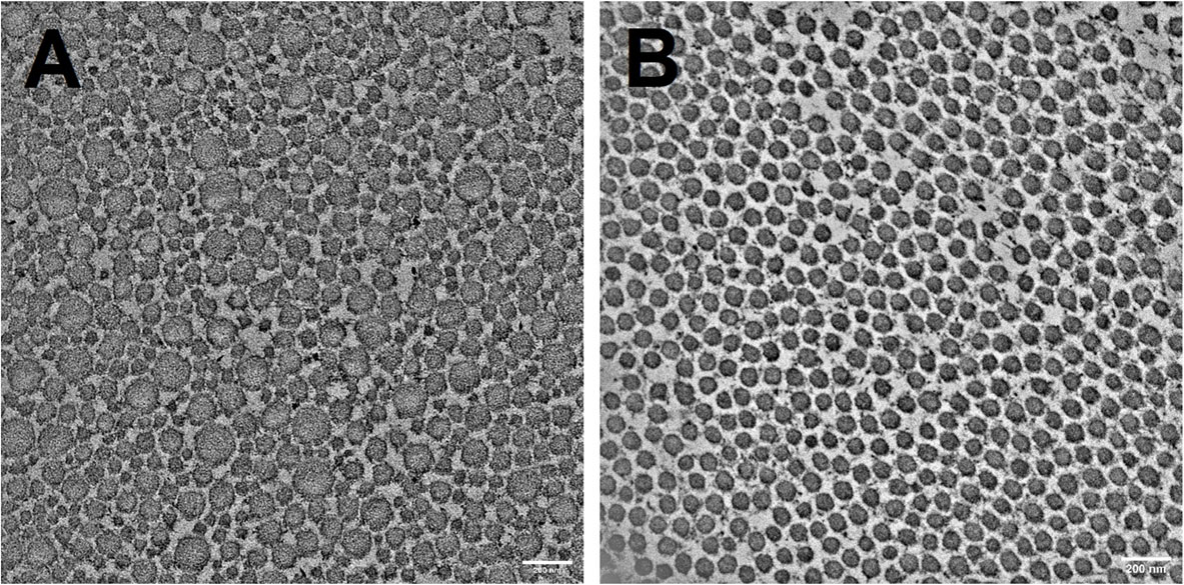
**a** and **b** Representative transmission electron microscopy images showing the fibril diameter composition in transversely sectioned tendons. The composition of the fibrils in the tendon from the fracture group (**a**) revealed a more heterogeneous fibril diameter distribution (mean 99.3 ± 42.4 nm) compared with the more homogeneous fibril distribution (mean 64.7 ± 7.1 nm) observed in the direct lateral revision group (**b**). Scale bar 200 nm

**Fig. 2:**
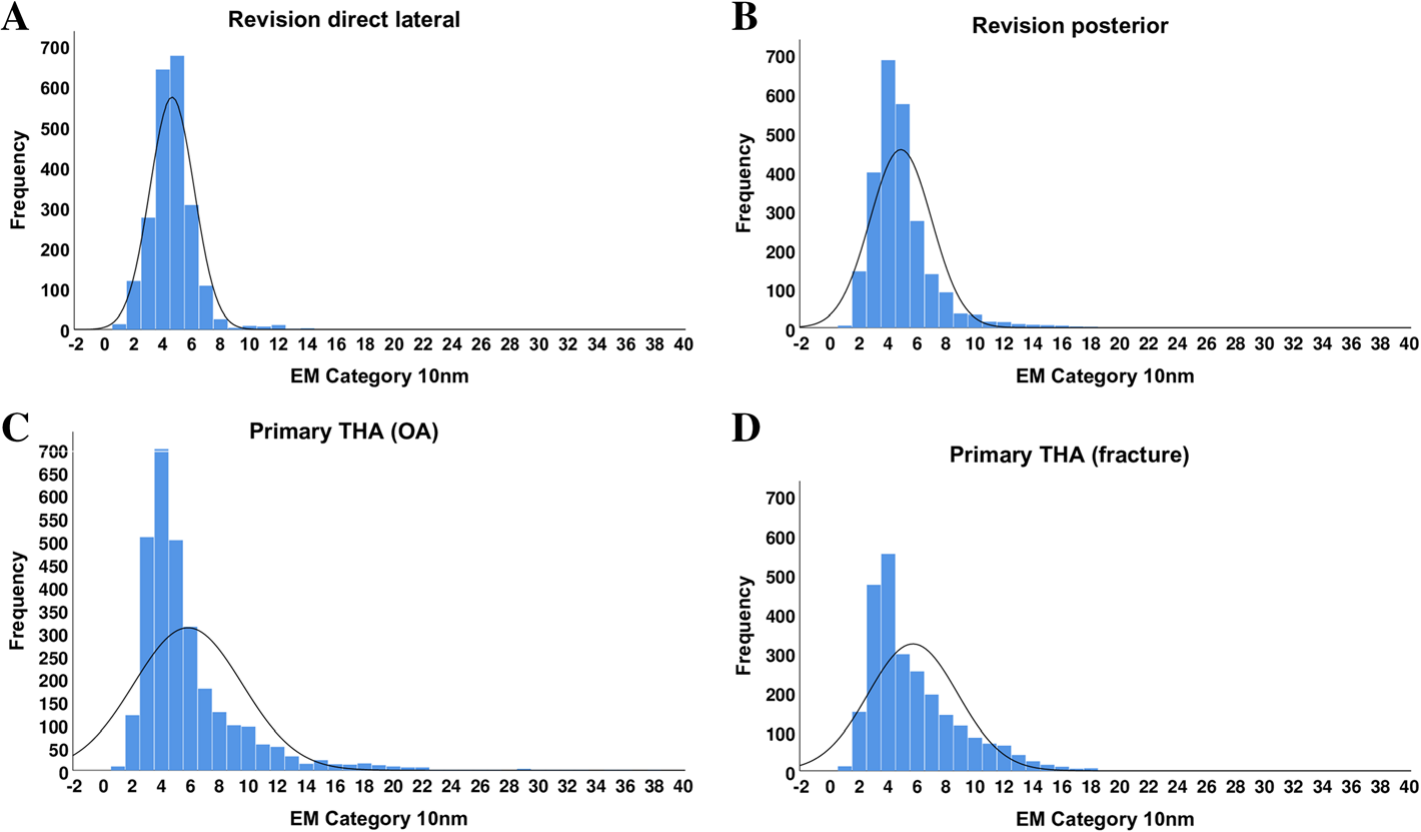
**a**–**d** Relative distribution of fibril diameters in the four study groups. Both the primary THA and the fracture groups show a wider range of fibril diameter than the revision groups

Both the direct lateral revision and posterior revision groups had statistically significantly smaller mean fibril diameters compared with the fracture and primary THA groups (*p* < 0.0001). Moreover, the direct lateral revision group had a statistically significantly smaller mean fibril diameter compared with the posterior revision group (*p* < 0.0001) (Table 3, Figs. 1 and 2a–d).

**Table 3:** Fibril diameter as seen in the TEM

GMED tendon fibrils	Revision DL	Revision posterior	Primary THA (OA)	Primary THA (fracture)	ANOVA
*N*	2201	2425	2900	2500	
Diameter in nm					***p*** **< 0.0001**
Mean (SD)	56.5 (15.3)	58.7 (21.2)	68.3 (37.3)	67.6 (30.9)	
Median	60.0	50.0	60.0	57.0	

Revision DL: revision arthroplasty via the direct lateral approach; Revision posterior: revision arthroplasty via the posterior approach; Primary THA (OA): primary total hip arthroplasty due to osteoarthritis; Primary THA (fracture): primary total hip arthroplasty due to femoral neck fracture. *N*, number of fibrils in areas showing transversely sectioned collagen fibrils; significant *p* value in bold. Pair-wise comparison showed statistical significance of *p* < 0.001 except for primary THA vs fracture (*p* = 0.43)

No significant difference in fibril diameter was found between the fracture and primary THA group (Table 3, Fig. 2c–d).

Data were missing in one biopsy sample in the primary THA group, due to the absence of structurally defined fibers in the biopsy.

### Histological evaluation

**Fig. 3:**
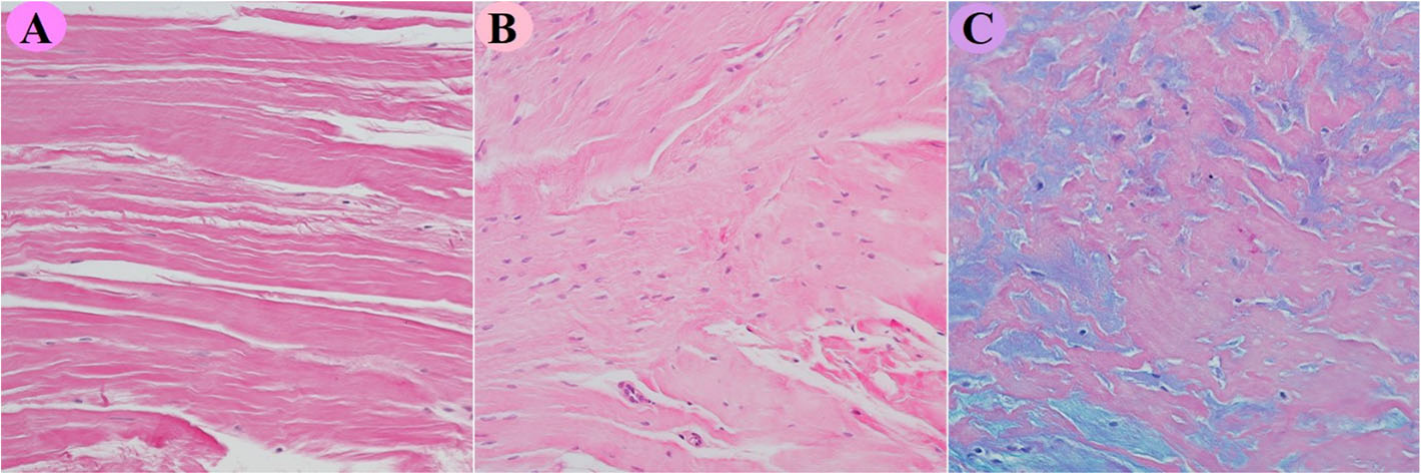
**a**–**c** Light-microscopic views of specimens obtained from three different patients from the gluteus medius tendon or tendon-like repair tissue. The a, b, and c views have a magnification of approximately ×100. The staining is hematoxylin and eosin in a and b and Alcian blue-PAS in c. Biopsy specimen A was obtained from a 61-year-old female patient who had had previous hip surgery performed with a posterior approach. The tendon-like tissue depicted parallel bundles of dense connective tissue with slight waviness and elongated tendon fibroblasts. Biopsy specimen B came from a 66-year-old male patient with previous hip surgery with a direct lateral approach and revealed dense collagen tissue with the derangement of bundle structure, increased cellularity with tendon fibroblasts with a somewhat rounded appearance and vessels within the tendon. Biopsy specimen C was obtained from a 64-year-old female patient with previous surgery via a direct lateral approach. The photomicrograph showed areas of blue alcianophilia corresponding to an increased content of glycosaminoglycans in the tendon-like gluteus medius tissue.

The distribution of the histological findings for the four elements of the TDS is reported in Table 4. The direct lateral revision group had a significantly higher total degeneration score (TDS) (Table 5) compared with the primary hip OA group (*p* = 0.004). There were missing data due to the absence of structurally defined fibers or insufficient tissue specimens in 7 samples in the posterior revision group, 7 samples in the primary THA group, one sample in the fracture group, and one sample in the direct lateral revision group. In addition, one TDS value was missing in the primary THA group. An example of histological findings is shown in Fig. 3a–c.

**Table 4:** Distribution of the four elements in the TDS

		Revision DL (*n* = 44)	Revision posterior (*n* = 48)	Primary THA (OA) (*n* = 58)	Primary THA (fracture) (*n* = 50)
Fiber structure	0	1 (2.3)	9 (18.8)	14 (24.1)	8 (16.0)
	1	12 (27.3)	12 (25.0)	16 (27.6)	16 (32.0)
	2	22 (50)	20 (41.7)	19 (32.8)	23 (46.0)
	3	8 (18.2)	-	2 (3.4)	2 (4.0)
Cellularity	0	4 (9.1)	14 (29.2)	32 (55.2)	12 (24.0)
	1	21 (47.7)	18 (37.5)	15 (25.9)	20 (40.0)
	2	10 (22.7)	8 (16.7)	4 (6.9)	14 (28.0)
	3	8 (18.2)	1 (2.1)	-	3 (6.0)
Vascularity	0	11 (25)	17 (35.4)	25 (43.1)	14 (28.0)
	1	18 (40.9)	17 (35.4)	14 (24.1)	25 (50.0)
	2	10 (22.7)	6 (12.5)	10 (17.2)	10 (20.0)
	3	4 (9.1)	1 (2.1)	2 (3.4)	-
GAGs	0	15 (34.1)	12 (25.0)	21 (36.2)	15 (30.0)
	1	14 (38.8)	12 (25.0)	20 (34.5)	25 (50.0)
	2	5 (11.4)	12 (25.0)	9 (15.5)	9 (18.0)
	3	9 (20.5)	5 (10.4)	-	-
Missing values (%)		1 (2.3)	7 (14.6)	7 (12.1)	1 (2.0)

Revision DL: revision arthroplasty via the direct lateral approach; Revision posterior: revision arthroplasty via the posterior approach; Primary THA (OA): primary total hip arthroplasty due to osteoarthritis; Primary THA (fracture): primary total hip arthroplasty due to femoral neck fracture

**Table 5:** The total degeneration score (TDS)

	Revision DL (*n* = 22)	Revision posterior (*n* = 22)	Primary THA (OA) (*n* = 27)	Primary THA (fracture) (*n* = 25)
TDS				
Mean (SD)	6.2 (2.4)*	4.9 (2.2)	4.0 (2.1)*	5.0 (2.0)
Median (range)	5.7 (3–11)	5.5 (1–8)	4.0 (0-7)	5.0 (0-7)
Missing values		2	2	

Rev DL: revision arthroplasty direct lateral approach; Rev post: revision arthroplasty posterior approach, Prim THA (OA): primary total hip arthroplasty due to osteoarthritis; Prim THA (fract): primary total hip arthroplasty due to femoral neck fracture.

Significant difference between Revision DL and Primary THA (OA), *p* = 0.004

## Discussion

The most important finding in the present study was that the ultrastructural evaluation revealed collagen fibrils with a smaller diameter in the GMED tendon of patients who had previously undergone THA, through either the posterior or direct lateral approach, compared with patients with primary OA of the hip and femoral neck fractures. Furthermore, the histological evaluation revealed more degeneration in the GMED tendon in the direct lateral revision group compared with the primary THA group.

To the authors’ knowledge, this is a rare study when it comes to comparing the histological and ultrastructural changes in a periarticular tendon in the hip of patients with and without hip OA, as well as in patients who had previously undergone THA through the direct lateral and posterior approaches.

Some studies in which MRI was used to evaluate the GMED tendon and muscle have revealed similar findings. Fatty atrophy and tendon damage to the gluteus minimus muscle and the lateral portion of the GMED tendon have been found after the direct lateral approach. Moreover, fatty atrophy and tendon damage to the external rotator muscles have been found after THA through the posterior approach [23]. In addition, abductor tendon defects and fatty atrophy of the GMED muscle and the posterior part of the GMED muscle, shown by MRI, were more common in patients with pain at the greater trochanter, who were limping or had gluteal weakness 1 year after THA compared with an asymptomatic control group [24]. Fatty atrophy of the GMED muscle was almost exclusively present in the symptomatic patients [25]. Moreover, fatty degeneration of the GMED increased after multiple revision THRs. A similar pattern has been found after primary THR but with considerably less muscle damage [26].

When the revision groups in the present study were compared, more ultrastructuralchanges indicating GMED tendon degeneration were found in the GMED tendon in patients in the direct lateral revision group compared with the posterior revision group. Both surgical splitting of the GMED tendon and a possible injury to the inferior branch of the superior gluteal nerve, the main nerve supplying the abductor muscles of the hip, during THA may affect the GMED muscle function negatively [27, 28].

Interestingly, injury to the hip abductors after THA via the posterior approach has recently been reported. In addition, more degenerative changes in the GMED have been reported on MRI after THA via the posterior approach compared with the direct lateral approach [29].

The present study did not reveal any ultrastructural difference between the GMED tendon in the fracture and primary THA groups, as expected. Histologically, the fracture group did not show a significantly lower TDS compared with any of the other groups. This could be caused by the acute inflammatory reaction associated with the bleeding in patients with femoral neck fractures. The relatively small size of these groups might also play a role in not showing the expected differences.

Like the result of the present study, no significant ultrastructural and histological differences in the subscapularis and hamstring tendons were found in patients with primary OA of the shoulder and knee respectively compared with control groups [15, 16].

The strengths of the study include the fact that the biopsies were obtained from living humans. The limitations of the study include the fact that it might be under-powered, despite the inclusion of 100 patients, as well as the fact that a non-optimal group of patients with femoral neck fractures undergoing THA served as controls, because it was ethically impossible to obtain GMED tendon biopsies from healthy age-matched individuals. The main reason for inclusion of the patients in the revision groups was due to loosening of the hip prosthesis, but in three patients due to the failure of metal-on-metal hip resurfacing arthroplasty or recurrent hip prosthesis dislocation. This could be a confounding variable as one would expect that repeated dislocations or wear debris may result in greater histological or ultrastructural changes as compared with primary THA. A delta of 5 nm in the mean fibril diameter was considered “meaningful” in the power analysis, despite that the authors have no evidence that 5 nm has a clinical effect.

The present study indicates that GMED tendon degeneration and subsequent rupture are more probable after THA was performed through the direct lateral approach than after the posterior approach. This might have an important clinical implication as it may explain why some patients continue to suffer from residual trochanteric pain and/or limping after THA, despite the hip prosthesis components being well positioned and aligned. The study also theoretically emphasizes the importance of appropriate rehabilitation after THA, aimed at strengthening the abductor and rotator muscles of the hip joint.

## Conclusions

The GMED tendon shows more degeneration in patients who undergo hip revision arthroplasty than in patients with primary OA of the hip and control patients, who have suffered a femoral neck fracture. Furthermore, patients who had previously undergone primary THA through a direct lateral approach revealed more histological GMED tendon degeneration than patients who suffer primary hip OA.

## Supplementary Information

**Additional file 1.** Histology original data

**Additional file 2.** Electron microscopy original data.

## Data Availability

The datasets generated and/or analyzed during the current study are not publicly available because individual privacy could be compromised, but are available from the corresponding author on reasonable request.

## Abbreviations

GMED: Gluteus medius
OA: Osteoarthritis
SD: Standard deviation
TEM: Transmission electron microscope
THA: Total hip arthroplasty
TDS: Total degeneration score
VAS: Visual analogue scale
